# Supplementary material for: Selection pressure on the rhizosphere microbiome can alter nitrogen use efficiency and seed yield in Brassica rapa
Source: Commun Biol. 2022 Sep 14;5:959. doi: 10.1038/s42003-022-03860-5 (PMC9474469; doi:10.1038/s42003-022-03860-5)
Supplement: Supplementary file 2 — Supplementary Information [file 42003_2022_3860_MOESM2_ESM.pdf]

Supplementary Information

Title: Selection pressure on the rhizosphere microbiome can alter nitrogen use efficiency and seed yield in *Brassica rapa*

Authors: Joshua Garcia[1], Maria Gannett[1], LiPing Wei[1], Liang Cheng[1], Shengyuan Hu[2], Jed Sparks[3], James Giovannoni[4], and Jenny Kao-Kniffin[1]*

Affiliations:

[1]School of Integrative Plant Science, Cornell University, Ithaca, NY, USA

[2]Machine Learning Department, Carnegie Mellon University, Pittsburgh, PA, USA

[3]Department of Ecology and Evolutionary Biology, Cornell University, Ithaca, NY, USA

[4]USDA-ARS and Boyce Thompson Institute, Ithaca, NY, USA

*Corresponding Author:

Jenny Kao-Kniffin, 135 Plant Science Building, Cornell University, Ithaca, NY 14853, E-mail: jtk57@cornell.edu, Phone: 607.255-8886, Fax: 607.255.0599

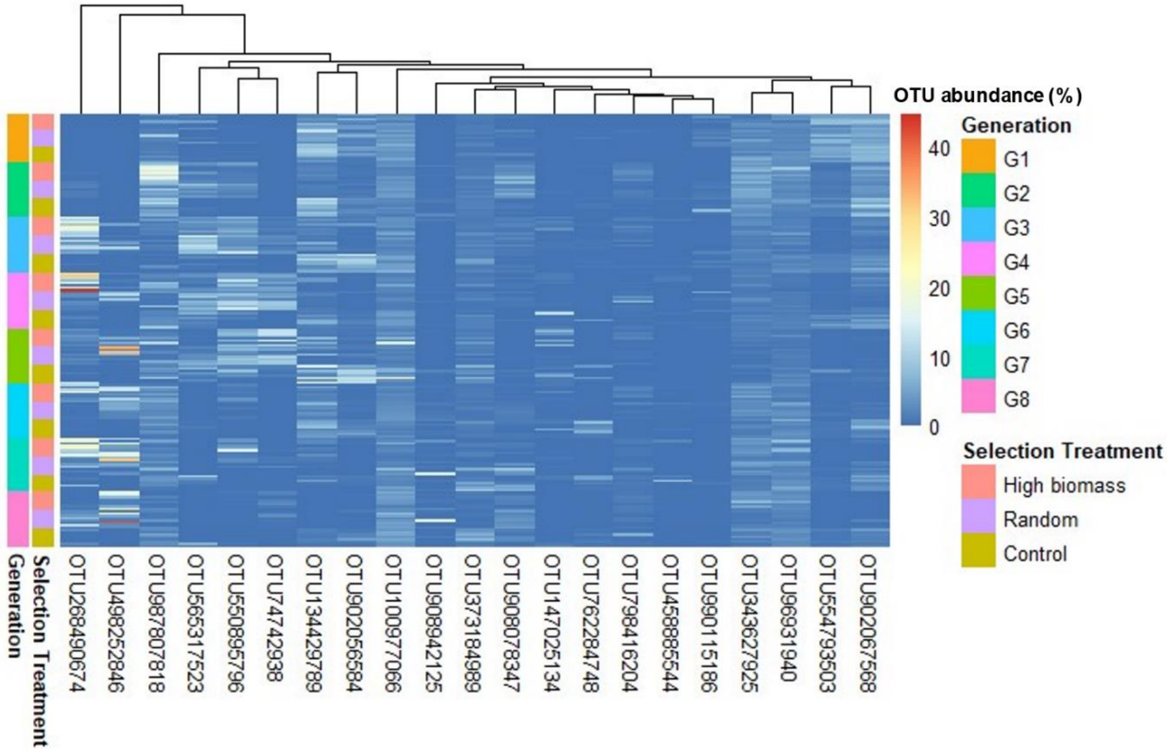

Supplementary Figure 1. Heatmap displaying shifts in the relative abundances of different bacterial taxa (OTUs) in each selection treatment for the 1st through 8th generation. Samples are grouped first by selection treatment then by generation as shown on the left side of the heatmap. Only OTUs with a maximum abundance greater than 7.5% are included in the heatmap, which was an adequate threshold to observe shifts in abundance. The color gradient (dark to light blue and light to dark red) represents increasing relative abundances (%) for each OTU in a given sample. Generation x Selection Treatment groups primarily had eight replicates each. In total, sequences from 186 soil samples were used in the analysis.

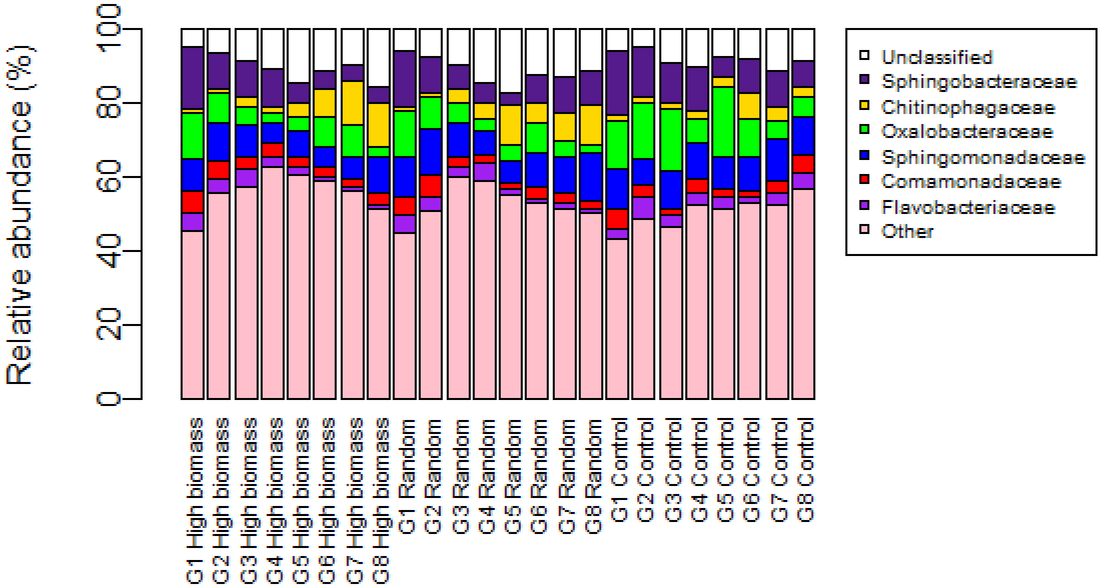

Supplementary Figure 2. Relative abundance (%) plots for every Generation x Selection Treatment group using the seven most abundant bacterial families. Each color on the bar represents the bacterial family average relative abundance (%) in a given Generation x Selection Treatment grouping for the 1st to 8th generations. Samples are grouped on the x axis by selection treatment then by generation. Generation x Selection Treatment groups primarily had eight replicates each.

a

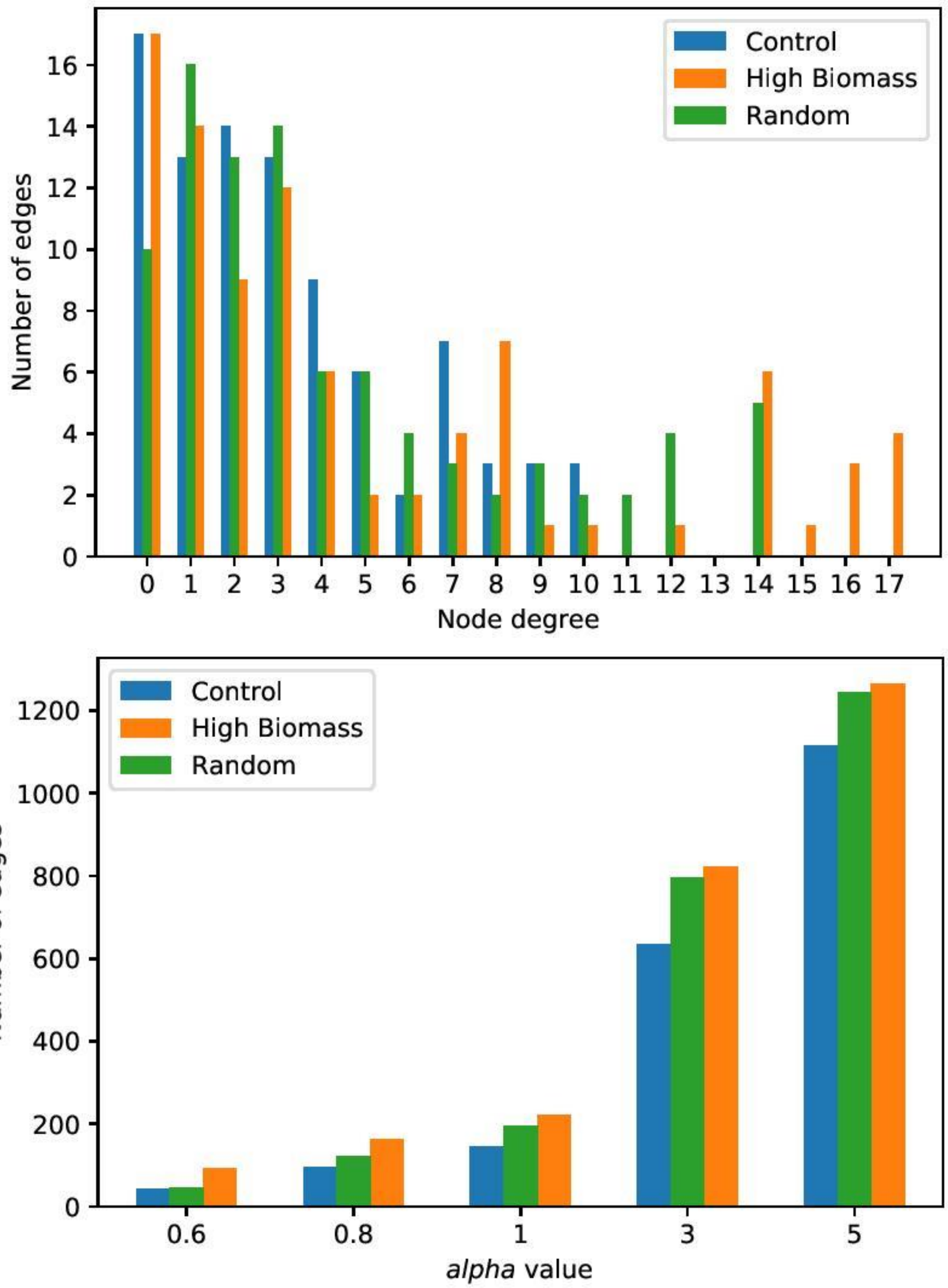

Supplementary Figure 3. Network analysis components for nodes and edges. **a** Bar plot displaying the distribution of degree of all nodes used in the analysis with sequences from all Generation x Selection Treatment groups. **b** Bar plot representing the total number of edges for each selection treatment given different α value thresholds using sequence data from all Generation x Selection Treatment groups. Each Generation x Selection Treatment group had at least seven replicates. In both graphs, the orange bars represent high biomass, blue bars represent random, and green bars represent the control.

Supplementary Table 1. Summary of the algorithm used to perform the extended local similarity analysis. *O* represents OTU time series data.

Data: O_1, O_2, time delay D
Result: local similarity score between O_1 and O_2
Initialize $P, N \in \mathbb{R}^{n\times n}$
for i in $[0, n-1]$ **do**
  $P_{i,0}, P_{0,i}, N_{i,0}, N_{0,i} = 0$
end
for i, j in $[0, n-1]$ with $|i-j| \leq D$ **do**
  $P_{i+1,j+1} = \max(0, P_{i,j} + O_{1,i+1}O_{2,j+1})$
  $N_{i+1,j+1} = \max(0, N_{i,j} - O_{1,i+1}O_{2,j+1})$
end
$P(O_1, O_2) = \max_{0\leq i,j<n} P_{i,j}$
$N(O_1, O_2) = \max_{0\leq i,j<n} N_{i,j}$
return $LS(O_1, O_2) = \frac{\max(P(O_1,O_2),N(O_1,O_2))}{n}$
