# Supplementary material for: Selection pressure on the rhizosphere microbiome can alter nitrogen use efficiency and seed yield in Brassica rapa
Source: Commun Biol. 2022 Sep 14;5:959. doi: 10.1038/s42003-022-03860-5 (PMC9474469; doi:10.1038/s42003-022-03860-5)
Supplement: Supplementary file 3 — Description of Additional Supplementary Data [file 42003_2022_3860_MOESM3_ESM.pdf]

Description of Additional Supplementary Files

File name: Supplementary Data 1
Description: The source data behind Figures 2 and 5 in the main manuscript.
