# Supplementary material for: Selection pressure on the rhizosphere microbiome can alter nitrogen use efficiency and seed yield in Brassica rapa
Source: Commun Biol. 2022 Sep 14;5:959. doi: 10.1038/s42003-022-03860-5 (PMC9474469; doi:10.1038/s42003-022-03860-5)
Supplement: Supplementary file 5 — Reporting Summary [file 42003_2022_3860_MOESM5_ESM.pdf]

nature portfolio

Corresponding author(s): Jenny Kao-Kniffin

Last updated by author(s): 6/30/2022

Reporting Summary

Nature Portfolio wishes to improve the reproducibility of the work that we publish. This form provides structure for consistency and transparency in reporting. For further information on Nature Portfolio policies, see our Editorial Policies and the Editorial Policy Checklist.

Please do not complete any field with "not applicable" or n/a. Refer to the help text for what text to use if an item is not relevant to your study.
For final submission: please carefully check your responses for accuracy; you will not be able to make changes later.

Statistics

For all statistical analyses, confirm that the following items are present in the figure legend, table legend, main text, or Methods section.

n/a	Confirmed	
☐	☒	The exact sample size (*n*) for each experimental group/condition, given as a discrete number and unit of measurement
☐	☒	A statement on whether measurements were taken from distinct samples or whether the same sample was measured repeatedly
☐	☒	The statistical test(s) used AND whether they are one- or two-sided<br>*Only common tests should be described solely by name; describe more complex techniques in the Methods section.*
☒	☐	A description of all covariates tested
☐	☒	A description of any assumptions or corrections, such as tests of normality and adjustment for multiple comparisons
☐	☒	A full description of the statistical parameters including central tendency (e.g. means) or other basic estimates (e.g. regression coefficient) AND variation (e.g. standard deviation) or associated estimates of uncertainty (e.g. confidence intervals)
☐	☒	For null hypothesis testing, the test statistic (e.g. *F*, *t*, *r*) with confidence intervals, effect sizes, degrees of freedom and *P* value noted<br>*Give P values as exact values whenever suitable.*
☒	☐	For Bayesian analysis, information on the choice of priors and Markov chain Monte Carlo settings
☒	☐	For hierarchical and complex designs, identification of the appropriate level for tests and full reporting of outcomes
☐	☒	Estimates of effect sizes (e.g. Cohen's *d*, Pearson's *r*), indicating how they were calculated

Our web collection on statistics for biologists contains articles on many of the points above.

Software and code

Policy information about availability of computer code

Data collection	No custom software was used to collect data in this experiment.
Data analysis	We used a custom code to run the Extended Local Similarity Analysis (eLSA) discussed in this paper. The code can be accessed at doi.org: 10.5281/zenodo.6800595

For manuscripts utilizing custom algorithms or software that are central to the research but not yet described in published literature, software must be made available to editors and reviewers. We strongly encourage code deposition in a community repository (e.g. GitHub). See the Nature Portfolio guidelines for submitting code & software for further information.

Data

Policy information about availability of data

All manuscripts must include a data availability statement. This statement should provide the following information, where applicable:

- Accession codes, unique identifiers, or web links for publicly available datasets
- A description of any restrictions on data availability
- For clinical datasets or third party data, please ensure that the statement adheres to our policy

The raw bacterial sequence dataset generated and analyzed during the current study are available in the National Center for Biotechnology Information repository, https://www.ncbi.nlm.nih.gov/sra/PRJNA833111. Additionally, we have provided a Supplementary Data file containing the data used to create figure 2 as requested by the editor. Additional datasets generated and during this experiment are available from the corresponding author on request.

Field-specific reporting

Please select the one below that is the best fit for your research. If you are not sure, read the appropriate sections before making your selection.

☒ Life sciences ☐ Behavioural & social sciences ☐ Ecological, evolutionary & environmental sciences

For a reference copy of the document with all sections, see nature.com/documents/nr-reporting-summary-flat.pdf

Life sciences study design

All studies must disclose on these points even when the disclosure is negative.

Sample size	We chose to cultivate 5 Brassica rapa plants in each individual pot in this experiment given Brassica plants have highly variable biomass production. Including multiple plants in a single pot helped to control for some of this variability in our phenotypic analyses. Each selection treatment had 15 replicate pots in every generation, which was also meant to control for highly variable Brassica rapa growth.
Data exclusions	In our sequencing data, we excluded samples that had poor PCR amplification, which is standard in soil microbiome studies. All other data gathered in this experiment was used in our statistical analyses following quality checks.
Replication	To allow for reproducibility of our findings, we have uploaded all bacterial sequencing data to NCBI SRA and the custom codes used for our Extended Local Similarity Analysis to GitHub. As indicated in our data availability statement, the corresponding author will also provide other data from the experiment on reasonable request.
Randomization	Each experimental pot in this experiment was randomly assigned to one of our three treatment groups.
Blinding	Blinding was not relevant to this study given our experimental units were potted plants.

Behavioural & social sciences study design

All studies must disclose on these points even when the disclosure is negative.

Study description	*NA*
Research sample	*NA*
Sampling strategy	*NA*
Data collection	*NA*
Timing	*NA*
Data exclusions	*NA*
Non-participation	*NA*
Randomization	*NA*

Ecological, evolutionary & environmental sciences study design

All studies must disclose on these points even when the disclosure is negative.

Study description	*NA*

Research sample | *NA*

Sampling strategy | *NA*

Data collection | *NA*

Timing and spatial scale | *NA*

Data exclusions | *NA*

Reproducibility | *NA*

Randomization | *NA*

Blinding | *NA*

Did the study involve field work? ☐ Yes ☑ No

Field work, collection and transport

Field conditions | *NA*

Location | *NA*

Access & import/export | *NA*

Disturbance | *NA*

Reporting for specific materials, systems and methods

We require information from authors about some types of materials, experimental systems and methods used in many studies. Here, indicate whether each material, system or method listed is relevant to your study. If you are not sure if a list item applies to your research, read the appropriate section before selecting a response.

Materials & experimental systems

n/a	Involved in the study	
☒	☐	Antibodies
☒	☐	Eukaryotic cell lines
☒	☐	Palaeontology and archaeology
☒	☐	Animals and other organisms
☒	☐	Human research participants
☒	☐	Clinical data
☒	☐	Dual use research of concern

Methods

n/a	Involved in the study	
☒	☐	ChIP-seq
☒	☐	Flow cytometry
☒	☐	MRI-based neuroimaging

Antibodies

Antibodies used | *NA*

Validation | *NA*

Eukaryotic cell lines

Policy information about cell lines

Cell line source(s)	NA
Authentication	NA
Mycoplasma contamination	NA
Commonly misidentified lines (See ICLAC register)	NA

Palaeontology and Archaeology

Specimen provenance	NA
Specimen deposition	NA
Dating methods	NA

☐ Tick this box to confirm that the raw and calibrated dates are available in the paper or in Supplementary Information.

Ethics oversight	NA

Note that full information on the approval of the study protocol must also be provided in the manuscript.

Animals and other organisms

Policy information about studies involving animals; ARRIVE guidelines recommended for reporting animal research

Laboratory animals	NA
Wild animals	NA
Field-collected samples	NA
Ethics oversight	NA

Note that full information on the approval of the study protocol must also be provided in the manuscript.

Human research participants

Policy information about studies involving human research participants

Population characteristics	NA
Recruitment	NA
Ethics oversight	NA

Note that full information on the approval of the study protocol must also be provided in the manuscript.

Clinical data

Policy information about clinical studies

All manuscripts should comply with the ICMJE guidelines for publication of clinical research and a completed CONSORT checklist must be included with all submissions.

Clinical trial registration	NA

Study protocol | NA

Data collection | NA

Outcomes | NA

Dual use research of concern

Policy information about dual use research of concern

Hazards

Could the accidental, deliberate or reckless misuse of agents or technologies generated in the work, or the application of information presented in the manuscript, pose a threat to:

No	Yes	
☒	☐	Public health
☒	☐	National security
☒	☐	Crops and/or livestock
☒	☐	Ecosystems
☒	☐	Any other significant area

Experiments of concern

Does the work involve any of these experiments of concern:

No	Yes	
☒	☐	Demonstrate how to render a vaccine ineffective
☒	☐	Confer resistance to therapeutically useful antibiotics or antiviral agents
☒	☐	Enhance the virulence of a pathogen or render a nonpathogen virulent
☒	☐	Increase transmissibility of a pathogen
☒	☐	Alter the host range of a pathogen
☒	☐	Enable evasion of diagnostic/detection modalities
☒	☐	Enable the weaponization of a biological agent or toxin
☒	☐	Any other potentially harmful combination of experiments and agents

ChIP-seq

Data deposition

☐ Confirm that both raw and final processed data have been deposited in a public database such as GEO.

☐ Confirm that you have deposited or provided access to graph files (e.g. BED files) for the called peaks.

Data access links
May remain private before publication. | *NA*

Files in database submission | *NA*

Genome browser session
(e.g. UCSC) | *NA*

Methodology

Replicates | NA

Sequencing depth | *NA*

Antibodies | *NA*

Peak calling parameters | *NA*

Data quality NA

Software NA

Flow Cytometry

Plots

Confirm that:

☐ The axis labels state the marker and fluorochrome used (e.g. CD4-FITC).

☐ The axis scales are clearly visible. Include numbers along axes only for bottom left plot of group (a 'group' is an analysis of identical markers).

☐ All plots are contour plots with outliers or pseudocolor plots.

☐ A numerical value for number of cells or percentage (with statistics) is provided.

Methodology

Sample preparation NA

Instrument NA

Software NA

Cell population abundance NA

Gating strategy NA

☐ Tick this box to confirm that a figure exemplifying the gating strategy is provided in the Supplementary Information.

Magnetic resonance imaging

Experimental design

Design type NA

Design specifications NA

Behavioral performance measures NA

Acquisition

Imaging type(s) NA

Field strength NA

Sequence & imaging parameters NA

Area of acquisition NA

Diffusion MRI ☐ Used ☐ Not used

Preprocessing

Preprocessing software NA

Normalization NA

Normalization template NA

Normalization template | NA

Noise and artifact removal | NA

Volume censoring | NA

Statistical modeling & inference

Model type and settings | NA

Effect(s) tested | NA

Specify type of analysis: ☐ Whole brain ☐ ROI-based ☐ Both

Statistic type for inference (See Eklund et al. 2016) | NA

Correction | NA

Models & analysis

n/a	Involved in the study
☒	☐ Functional and/or effective connectivity
☒	☐ Graph analysis
☒	☐ Multivariate modeling or predictive analysis

Functional and/or effective connectivity | NA

Graph analysis | NA

Multivariate modeling and predictive analysis | NA

This checklist template is licensed under a Creative Commons Attribution 4.0 International License, which permits use, sharing, adaptation, distribution and reproduction in any medium or format, as long as you give appropriate credit to the original author(s) and the source, provide a link to the Creative Commons license, and indicate if changes were made. The images or other third party material in this article are included in the article's Creative Commons license, unless indicated otherwise in a credit line to the material. If material is not included in the article's Creative Commons license and your intended use is not permitted by statutory regulation or exceeds the permitted use, you will need to obtain permission directly from the copyright holder. To view a copy of this license, visit http://creativecommons.org/licenses/by/4.0/
